# Thrombospondin 1 and 2 regulate mesenchymal progenitor cell fate and matrix organization

**DOI:** 10.1038/s41413-025-00493-2

**Published:** 2026-01-19

**Authors:** Madysen K. Hunter, Sneha Korlakunta, Neda Vishlaghi, Monisha Mittal, Kyle Cragg, Conan Juan, Chase A. Pagani, Yuxiao Sun, Lindsey Lammlin, Karen Kessell, Dylan Feist, Ji Hae Choi, Meng-Lun Hsieh, Jahnu Saikia, Craig L. Duvall, Heeseog Kang, Andrea I. Alford, Kurt D. Hankenson, Robert J. Tower, Tristan Maerz, Benjamin Levi

**Affiliations:** 1 Center for Organogenesis, Regeneration and Trauma, University of Texas Southwestern, Dallas, TX USA; 2 Department of Orthopedic Surgery, University of Michigan, Ann Arbor, MI USA; 3 Department of Biomedical Engineering, Vanderbilt University, Nashville, TN USA

**Keywords:** Pathogenesis, Bone

## Abstract

Thrombospondin 1 and 2 (TSP1 and TSP2) are critical regulators of extracellular matrix (ECM) interactions, influencing cell differentiation and tissue repair. Recent discoveries from our laboratory and others highlight the importance of altered ECM alignment in influencing aberrant mesenchymal progenitor cell (MPC) differentiation and subsequent ectopic bone formation in trauma-induced heterotopic ossification (HO). However, the key regulators of this MPC to ECM interaction have yet to be elucidated. This study uncovers the role of matricellular TSP1 and TSP2 in MPC/ECM interaction as well as HO formation and progression. Using single-cell RNA sequencing, spatial transcriptomics, and in vivo models, we found that TSP1 is upregulated in tissue remodeling macrophages and MPCs at the injury site, while TSP2 is restricted to MPCs surrounding the HO anlagen. TSP1/2 double knockout (DKO) mice exhibited significantly reduced HO volume and disrupted ECM alignment. These findings highlight the crucial roles of TSP1 and TSP2 in musculoskeletal injury repair as well as HO formation and progression, supporting the potential to therapeutically target TSP1 and TSP2 to prevent HO.

## Introduction

Heterotopic ossification (HO) is the atypical formation of bone in soft tissues, commonly arising from traumatic injuries, surgeries, or specific genetic conditions.^1^ This aberrant bone growth can cause chronic pain and hinder mobility significantly diminishing a patient’s quality of life.^2^ In the U.S., nearly 20% of primary joint replacement patients, 80% of revision hip replacement patients, and 20% of severe burn victims are at risk of developing HO.^2–7^ Over 90% of these cases manifest HO at the site of injury, surgery, or burn—areas abundant in extracellular matrix and where mesenchymal cells interact with the collagen matrix.^4–7^ We have established that traumatic HO necessitates injury, originates from mesenchymal progenitor cells (MPCs) and predominantly occurs at connective tissue sites involving collagen fibrillogenesis.^8,9^ Our previous findings highlight an intriguing prerequisite for HO: alterations in extracellular matrix (ECM) alignment. By inhibiting ECM alignment via injury site immobilization, we not only prevented HO onset but also observed a shift in mesenchymal progenitor cell (MPC) lineage commitment from osteogenesis to adipogenesis.^8^

Thrombospondins (TSPs) are matricellular glycoproteins that influence cell-to-cell and cell-to-matrix interactions.^10^ Thrombospondins interact with cell surface receptors and ECM proteins to regulate cytokine and growth factor bioavailability.^11^ Notably, TSPs are predominantly present in connective tissues during development and are reactivated post-injury, where they have roles in collagen fibrillogenesis in bone and non-osseous connective tissues.^12,13^ Macrophage-derived TSP1 has been shown to contribute to diseases including abdominal aortic aneurysms and pulmonary hypertension by mediating abnormal collagen deposition via matrix metalloproteinase (MMP) activation, nitric oxide inhibition, and TGF-β activation.^14^ Additionally, TSP1 is known to interact with CD36 and integrins on macrophages, influencing their adhesion, migration, and pro-inflammatory cytokine production.^15–19^ TSP2 is essential for osteoblast lineage progression through its modulation of MPCs. TSP2 plays a crucial role in the differentiation of MPCs into osteoblasts, which are vital for proper bone formation and remodeling. In the absence of TSP2, matrix collagen levels are significantly decreased with reductions in matrix mineralization and bone matrix quality.^13,20–22^

Given the role of TSPs in collagen properties and the ECM-MPC interactions, we performed single-cell transcriptomics, spatial transcriptomics, in vivo modulation of TSP1 and 2 signaling, micro-computed tomography, and second harmonic generation imaging to explore the role of TSP1 and 2 on ECM architecture and HO formation. Overall, our findings define the spatial and temporal expression pattern of *Thbs1* (TSP1) and *Thbs2* (TSP2) in trauma-induced HO and uncover the essential function of these proteins in ECM organization, osteogenic differentiation, and subsequent HO formation following musculoskeletal injury.

## Results

### *Thbs1* and *Thbs2* are upregulated following an HO inducing injury

**Fig. 1:**
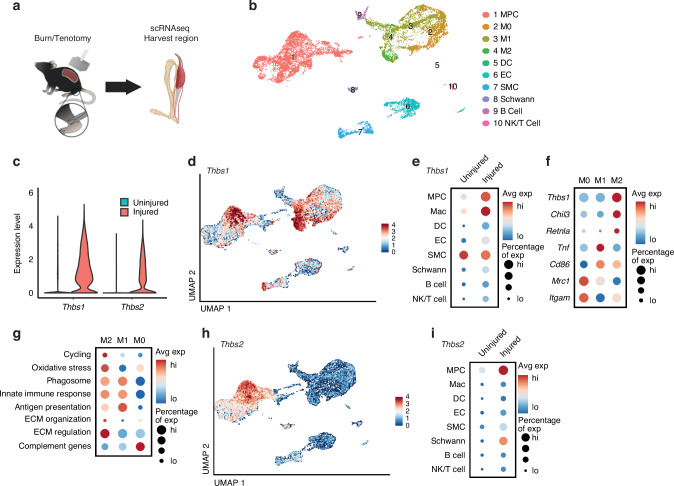
Mesenchymal progenitor cells (MPCs) and M2-like macrophages express TSP2 and TSP 1, respectively, after injury. **a** Schematic of burn/tenotomy injury site collected for single cell RNA sequencing (scRNAseq). **b** UMAP of cells collected from injury site 0,3,7, and 21 days after injury. **c** Violin plot of *Thbs1* and *Thbs2* from uninjured (day 0) mice and injured mice. **d** Feature plot of *Thbs1* expression. **e** Dot plot of *Thbs1* expression in uninjured and injured mice across all clusters. **f** Dot plot of *Thbs1* and macrophage markers for M0 (*Mrc1, Itgam*), M1(*Tnf, Cd86*), and M2(*Chil3, Retnla*). **g** Dot plot of *Thbs1* expression and macrophage paths markers enriched at the HO site. **h** Feature plot of *Thbs2* expression. **i** *Thbs2* expression in uninjured and injured mice across all clusters

To investigate the transcriptional changes occurring within the musculoskeletal injury site during HO formation and progression, we revisited previously published single-cell RNA sequencing (scRNA-seq) datasets generated in our laboratory utilizing a well-characterized mouse model involving a burn/tenotomy (B/T) injury where mice undergo a 30% total body surface area dorsal burn and full Achilles tendon transection without repair.^8,9,23–32^ Cells were harvested from the tendon injury site at four-time points (uninjured, 3, 7, and 21 days after injury) for analysis (GSE12606) (Fig. 1a). To explore the potential differential expression of genes involved in ECM signaling, we examined the expression of TSP1 and TSP2 (*Thbs1* and *Thbs2*). Our findings demonstrate significant upregulation of *Thbs1* and *Thbs2* in response to a B/T injury, underscoring their potential roles in ECM signaling during HO formation (Fig. 1b, c). In addition, to explore whether this thrombospondin signature is conserved in human HO, we reanalyzed a published microarray dataset (GSE94683) comparing bone marrow-derived MPCs from neurogenic HO patients to healthy donors.^33^ Notably, *Thbs1* and *Thbs2* were significantly upregulated in the HO derived MPCs compared to healthy donors (*THBS1* logFC = 0.892 521, *P* = 0.000 1; *THBS2* logFC= 0.844 618, *P* = 0.007 7, mirroring our murine results and highlighting a potential cross-species relevancy (Fig. S1C).

*Thbs1* transcripts were most highly expressed in macrophage populations at the injury site, with the highest levels observed three days after injury (Figs. 1d, e and S1B–E), consistent with the time point with the highest macrophage population after injury.^24^ *Thbs1* expression was also detected in the MPC population, however, to a lesser extent. Within the macrophage subpopulations defined through MacSpectrum,^34^ *Thbs1* expression was highest among M2-like anti-inflammatory macrophages (Fig. 1f). These macrophages are known for their capacity to promote tissue regeneration and ECM remodeling, both crucial during HO formation.^1,24,32^ They produce key cytokines, including TGF-β1 and VEGF-A, which facilitate MPC proliferation and differentiation into osteoblasts, essential for new bone formation.^1,24,32^ As recent studies have shown, macrophages do not simply conform to a static M1/M2 classification but instead exist along a flexible spectrum of activation states that are dynamically influenced by tissue-specific cues and inflammatory conditions.^35^ Within this spectrum, *Thbs1* is particularly associated with the remodeling path (ECM organization and regulation), playing a pivotal role in ECM remodeling^35^ (Fig. 1g). When comparing *Thbs1* expression among bone marrow, blood, and injury site cell populations 3 and 7 days after injury, the highest expression of *Thbs1* was observed at the injury site (Fig. S1D–F). Specifically, *Thbs1* expression was markedly elevated in tissue-resident macrophages at the injury site, suggesting a local upregulation of *Thbs1* in response to injury. While circulating macrophages present in the blood also exhibited *Thbs1* expression, the levels were considerably lower than levels observed at the injury site (Fig. S1G). *Thbs1* expression was further decreased within macrophages from the bone marrow (Fig. S1G). These results suggest that *Thbs1* expression is activated after monocytes/macrophages mobilize into circulation rather than originating in the bone marrow. Furthermore, the highest levels of *Thbs1* expression were consistently found at the injury site, implying a crucial role of macrophages and possibly other local cells in upregulating *Thbs1* in the local tissue environment. Taken together, the expression of *Thbs1* in M2-like anti-inflammatory macrophages associated with ECM organization and remodeling at the site of HO suggests that TSP1 may play a role in resolving local inflammation and remodeling the ECM to support tissue repair and new bone formation at the injury site.

### *Thbs2* is expressed exclusively in MPCs related to fibrillogenesis surrounding the HO site

Immobilization is a commonly used therapy for patients after extremity trauma. It has been established that immobilization of the tenotomy site after B/T results in decreased HO volume. This immobilization also resulted in altered collagen matrix alignment and a consequent decrease in osteogenesis.^8^ Based on these previous findings, we next set out to examine the impact of immobilization on *Thbs2* expression given its role in collagen fibrillogenesis. Differential gene expression between immobilized and mobilized mice 0, 3, and 7 days after B/T injury reveals a significant downregulation of *Thbs2* in immobilized mice 7 days after injury (GSE150995) (Fig. S2A–C). Notably*, Thbs2* expression was most prominently downregulated in the MPC population of immobilized mice (Fig. S2D). *Thbs2* differential downregulation in immobilized mice suggests that mechanical forces may play a role in regulating *Thbs2* expression. Given the known roles of TSP2 in regulating ECM dynamics, inflammation, and cell differentiation, this observation prompted us to further investigate the expression and role of *Thbs2* over the course of HO development.

**Fig. 2:**
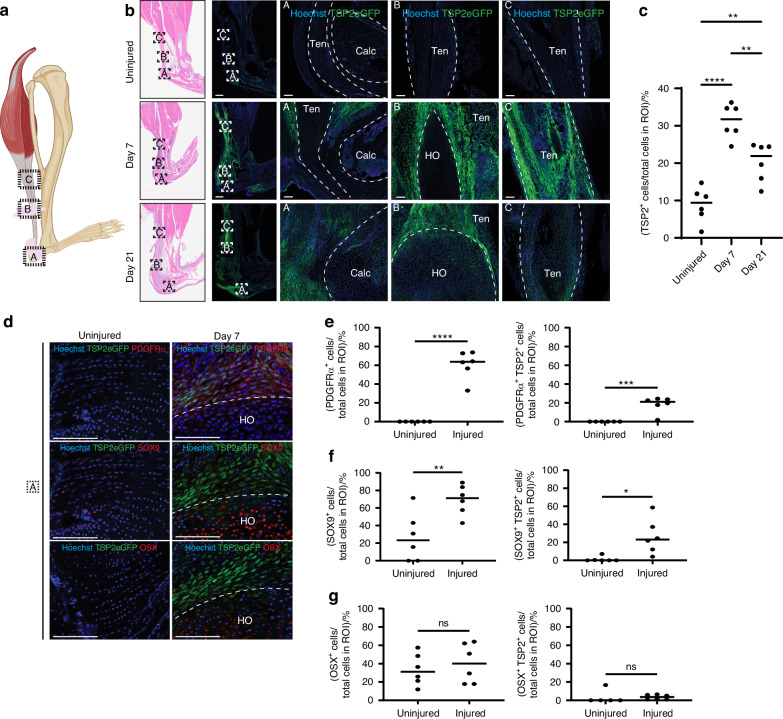
Temporal expression and localization of TSP2 following injury. **a** Schematic of HO anlagen after B/T injury; dashed boxes indicate IF region of interest (ROI). Letters correspond to magnified panels. **b** Representative tile scans and magnified regions from TSP2eGFP reporter mice at day 0, 7, and 21. White dashed lines outline anatomical areas (Ten = Achilles tendon, Calc = calcaneus, HO = HO anlagen). **c** Quantification of TSP2⁺ cells in ROI (percentage of all cells). Ordinary one-way ANOVA comparing uninjured, day 7, and day 21; *n* = 3 mice/group, 2–3 images/mouse. **d** IF of TSP2eGFP reporter mice at day 7 for PDGFRα, SOX9, and OSX. White dashed line = HO anlagen border. **e**–**g** Quantification of PDGFRα⁺, SOX9⁺, and OSX⁺ cells, and their overlap with TSP2⁺ cells, in uninjured vs day 7 mice (percentage of total cells in ROI). *n* = 3 mice/group, 2–3 images/mouse. ****P < 0.000 1, ***P < 0.001, **P < 0.01, *P < 0.05

Upon querying *Thbs2* transcripts in our dataset of cells harvested from the B/T injury site 0, 3, 7, and 21 days after injury, we found that *Thbs2* was almost exclusively and most highly expressed among the pre-HO progenitor population of MPCs (Fig. 1h, i). At day 7 after injury, *Thbs2* expression peaks in MPC populations (Fig. S1F). Given these scRNAseq-based insights in *Thbs2* expression, we next utilized a *TSP2-eGFP* reporter system to spatially characterize TSP2-eGFP expression at the HO site at timepoints leading up to the mineralization phase.^36^ Fluorescent microscopy demonstrated that *TSP2-eGFP* signal significantly increased on day 7 and 21 after injury around the HO site and tendon when compared to uninjured contralateral legs collected at the same time point (Figs. 2a, b and S3A–C). This *TSP2-eGFP* signal was highest on day 7, which is consistent with our scRNAseq findings of maximal *Thbs2* expression 7 days after injury (Fig. 2c). We confirmed the presence of TSP2^+^ MPCs at the injury site 1 week after injury by containing the HO injury site for the MPC marker platelet-derived growth factor receptor-alpha (PDGFRa) (Fig. 2d, e). We observed a significant percentage of PDGFRα^+^ TSP2^+^ cells at the HO injury site, consistent with our scRNA-seq findings that MPCs are a major source of TSP2 (Fig. 2d, e). To highlight the onset of endochondral ossification at the HO anlagen, the same set of samples were also stained for the chondrogenic transcription factor SRY-Box Transcription Factor 9 (SOX9) and the hypertrophic chondrocyte/osteoprogenitor marker Osterix (OSX). This staining revealed an increase in SOX9^+^ and dual SOX9^+^ TSP2^+^ cells in injured limbs 7 days after injury, consistent with the active process of endochondral formation and chondrocyte differentiation (Fig. 2d, f).^1^ OSX^+^ cells were also observed at the site with no significant difference between injured and uninjured limbs observed. Dual OSX^+^ TSP2^+^ cells were rarely observed (Fig. 2d, j). Our results show *Thbs2* is predominantly expressed in MPCs associated with HO formation, and TSP2 signal is appreciated around the HO anlagen, suggesting that TSP2^+^ MPCs are not only present, but likely contribute to the dynamic changes in the ECM environment required for bone formation.

### Spatial transcriptomics reveals *Thbs1* and *Thbs2* expression within and surrounding the HO anlagen, respectively

In musculoskeletal injuries, the spatial context of gene expression patterns is critically important to understand, given the substantial variability in function across anatomic regions. Therefore, we next utilized spatial transcriptomics using the Visium platform to characterize *Thbs1* and *Thbs2* expression at the tenotomy injury HO site.^27^

**Fig. 3:**
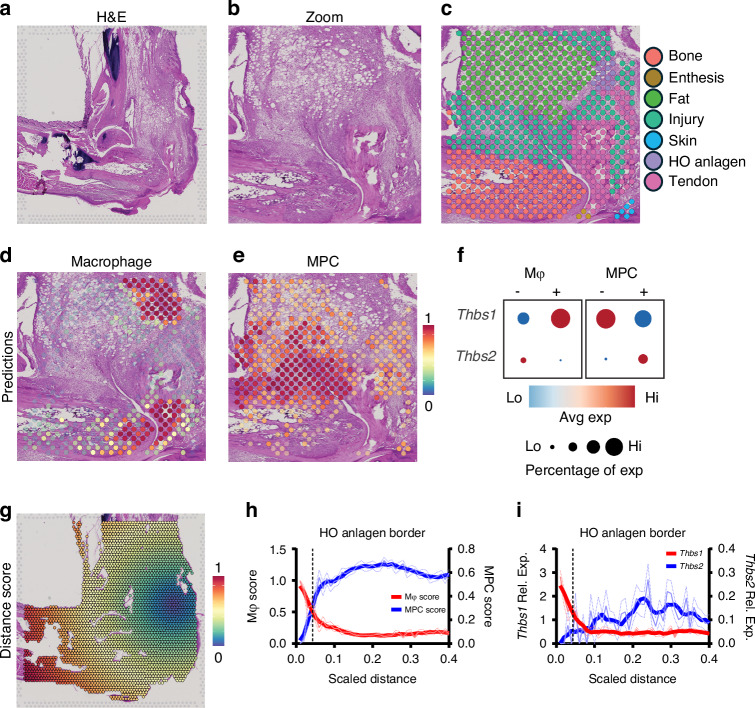
Spatial transcriptomic analysis reveals *Thbs1/2* expression patterns and cell type distributions relative to HO anlagen. **a** H&E histology section of injured leg day 7 after B/T injury. **b** Zoom representing magnified H&E region of interest (ROI). **c** H&E labeled for tendon, enthesis, injury, HO anlagen, bone, fat and skin. **d** Prediction scores for likelihood myeloid cells present in spatial dot. **e** Prediction scores for likelihood MPC cells present in spatial dot. **f** Dot plot of Thbs1 and Thbs2 across spatial dots defined as either macrophage (Mψ) and MPCs. **g** H&E superimposed with distance score from HO anlagen. **h** Spatialtime analysis of macrophage (Mψ) and MPC scores across scaled distance from HO site. Dashed line represents HO anlagen border. **i** Spatialtime analysis of *Thbs1* and *Thbs2* expression from HO anlagen. Dashed line represents HO anlagen border

We manually defined spatial spots to their corresponding tissue types on the H&E-stained histological slide using morphological landmarks^27^ (Fig. 3a–c). Leveraging our scRNAseq data, we set out to map the anatomical relationship of macrophage and MPC populations to the HO anlagen. Prediction scores, which correspond to the likelihood of macrophage or MPCs existing at each spot, were calculated (Fig. 3d, e). Those values were used to define cell-type positive or negative spots (i.e., MPC-positive vs MPC-negative) (Fig. S4A–F). Prediction scores for macrophage cells were highest at the HO anlagen and at the enthesis/tendon and bone regions. MPC prediction scores were high at the injury site but notably absent from the HO anlagen, where the beginning stages of endochondral ossification are observable. We then analyzed the expression patterns of *Thbs1* and *Thsb2* across these spots. As expected, given our scRNAseq findings, spots defined as positive for macrophage cells had a high expression of *Thbs1*, while MPC-positive spots were high for *Thbs2* expression (Fig. 3f).

To evaluate the spatial distribution of cell types within and around the HO anlagen, we analyzed cell type prediction scores in relation to SpatialTime, a distance-based metric where lower SpatialTime corresponds to spots closer in proximity to the HO anlagen^37^(Fig. 3g). Within the HO anlagen, we observed a high prediction score for macrophages and a low prediction score for MPCs. At the HO anlagen border, this relationship is reversed, and the likelihood of MPC presence significantly increases (Fig. 3h). This inverse distribution indicates the potential existence of a macrophage-MPC axis, where macrophages predominate at the HO anlagen, while MPCs largely exist at the perimeter. We also examined the relative expression levels of *Thbs1* and *Thbs2* across SpatialTime and observed a similar inverse expression pattern of the distribution of macrophage and MPCs. The relative expression of *Thbs1* was found to be highest at the HO anlagen, steadily decreasing as SpatialTime increased. Conversely, *Thbs2* showed limited expression at the HO anlagen, but increased significantly as SpatialTime increased, peaking before decreasing again (Fig. 3i). These results highlight distinct spatial patterns of *Thbs1* and *Thbs2* expression, with *Thbs1* concentrated at the HO anlagen with macrophages and *Thbs2* predominantly expressed in regions surrounding the injury with MPCs, suggesting specialized roles in local tissue remodeling and repair.

### TSP1 and 2 coordinate MPC fate and extracellular matrix remodeling following injury

**Fig. 4:**
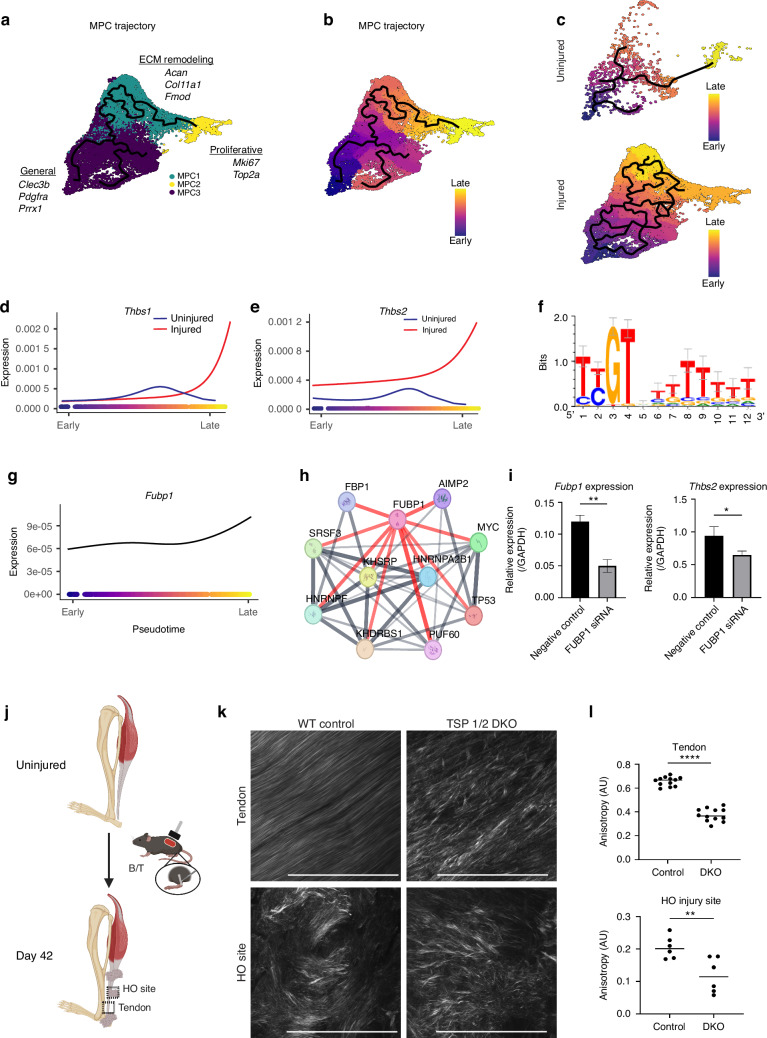
TSP1/2-driven ECM remodeling guides MPC differentiation. **a** Pseudotime clusters of MPCs (ECM remodeling, proliferative, general). **b** MPC pseudotime trajectory. **c** Uninjured vs. injured trajectories. **d**, **e** *Thbs1* & *Thbs2* regression in uninjured vs. injured MPCs. **f** FUBP1 binding motif. **g** *Fubp1* regression plot. **h** *Fubp1* regulatory network. **i** RTqPCR of MPCs transfected with control or *Fubp1* siRNA *Fubp1* & *Thbs2* expression (*Gapdh* control, 48 h). **j** Anatomical schematic of SHG sites. **k** SHG of collagen fibrils in tendon and HO from WT and DKO mice. **l** AU quantification: tendon *P* < 0.000 1, HO site *P* = 0.008 1 (*n* = 3/group)

To investigate the role of *Thbs1* and *Thbs2* in MPC differentiation during tendon injury response, we conducted a pseudotime analysis within the MPC niche using Monocle3. Along the trajectory, three distinct clusters were identified: General MPCs, ECM remodeling MPCs, and Proliferative MPCs (Fig. 4a, b). In uninjured tendon, MPCs predominantly progressed towards a proliferative terminal differentiation, reflecting a homeostatic state focused on tissue maintenance.^38^ Conversely, MPCs from injured tendons shifted their differentiation trajectory towards the ECM remodeling lineage (Fig. 4c). This shift indicates that injury prompts MPCs to engage in tissue repair processes following HO induction that involve significant ECM synthesis and remodeling.^39^ *Thbs1* and *Thbs2* expression across MPC trajectory demonstrates a striking divergence between uninjured and injured groups (Fig. 4d, e). In injured MPCs, both *Thbs1* and *Thbs2* exhibited pronounced upregulation, peaking at the latest stages of differentiation. In contrast, uninjured MPCs maintained a relatively stable and low expression levels of these genes throughout the trajectory.

Given that *Thbs2* is particularly enriched in MPCs, especially shortly after injury, we focused further analysis on *Thbs2* covariant module analysis using pseudotime to gain deeper insights into the transcriptional networks regulating *Thbs2* associated ECM remodeling in MPCs (Fig. S5C). The gene set derived from co-regulation network analysis identified genes associated with ECM organization and remodeling including *Col12a1, Col27a1, Plod2*, and *Mmp13* (Fig. S5D). Transcription factor (TF) motif analysis was performed to identify potential regulatory proteins that drive the coordinated expression of the identified gene set, providing insights into the upstream signals governing ECM remodeling. This analysis identified Far Upstream Element Binding Protein 1 (FUBP1) as a highly probable regulator of *Thbs2* in MPCs (Fig. 4f–h). Following siRNA-mediated *Fubp1* knockdown in MPCs derived from the tendon injury site, *Fubp1* transcript levels decreased by over 50%. In parallel, *Thbs2* expression was reduced by approximately 25% compared to negative control (Fig. 4i). To further explore this relationship, we examined RNA-seq data from *Fubp1*- knockout mice and a CRISPR-engineered RPE1 cell line carrying the cancer-associated A38D point mutation, disrupting FUBP1’s interaction with the splicing factor U2AF2 and impairs efficient splicing of long introns.^40^ In both models, *Thbs2* expression was significantly reduced, consistent with a broader role for FUBP1 in regulating *Thbs2* expression (Fig. S5E).

### Loss of TSP1 and 2 disrupts matrix structure dynamics and inhibits HO in vivo

To further investigate the importance of thrombospondin in MPC and macrophage-mediated ECM organization and HO development, we utilized TSP1 and TSP2 double knockout (TSP1/2 DKO) mice. Using age- and sex-matched C57BL/6 mice as wild-type (WT) controls, we conducted our B/T model on both genotypes and performed quantitative analyses of tissue organization and bone formation (Fig. 4j). Six weeks post-injury, we employed second harmonic generation (SHG) microscopy to visualize ECM collagen organization along the injured Achilles tendon and at the HO anlagen. The SHG microscopy revealed notable differences in collagen fibril organization between DKO and WT mice (Fig. 4k, l). In WT mice, collagen fibers were more aligned and organized with a higher anisotropy (AU), reflecting a structured ECM conducive to proper bone formation and tissue repair. In contrast, DKO mice displayed a more disorganized distribution of collagen fibrils, with less alignment demonstrated by a lower AU and more random orientation throughout the hindlimb (Fig. 4k, l). This disorganization suggests that TSP1 and TSP2 are critical in maintaining ECM integrity and alignment throughout the healing process, and when both TSP1 and TSP2 are absent, organization is significantly disrupted.

**Fig. 5:**
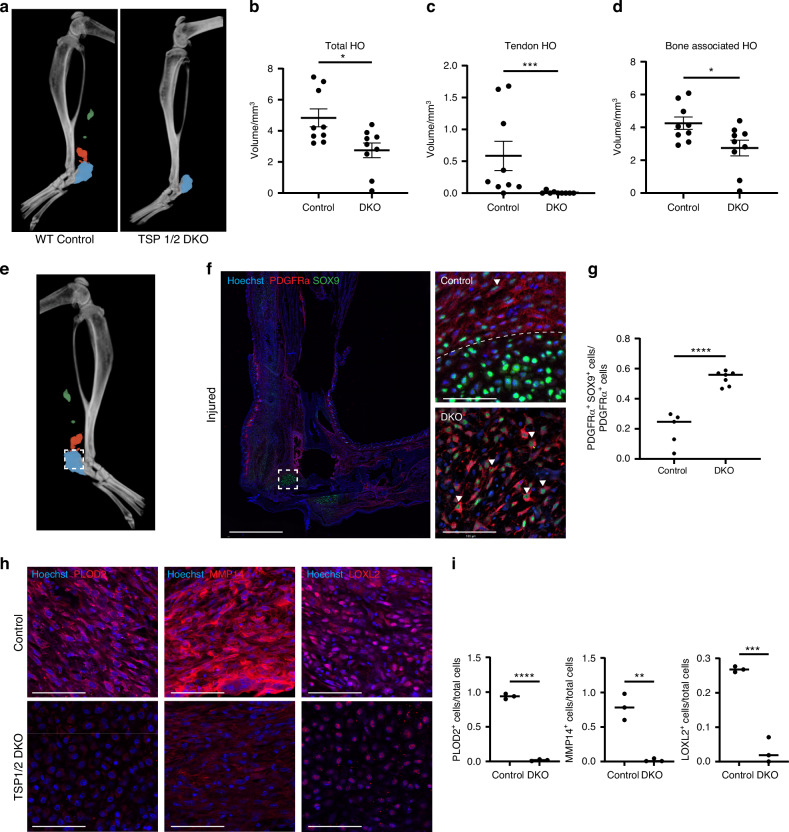
TSP1/2 deletion reduces HO volume and alters MPC distribution. **a** 3D micro-CT of WT and TSP1/2 DKO mice 9 weeks post–burn tenotomy (green = proximal tendon HO, red = distal tendon HO, blue = calcaneal HO). **b**–**d** Quantified HO volume: total (*P* = 0.013 0), tendon (*P* = 0.000 6), and bone-associated (*P* = 0.024 1); *n* = 9/group. **e** WT micro-CT with ROI (white box) shown in IF. **f** IF staining for Hoechst, PDGFRa, and SOX9 in control vs. DKO. **g** Quantification of PDGFRα^+^ SOX9^+^ cells/total PDGFRa^+^ cells (*P* < 0.000 1, *n* = 2–3/group). **h** IF of injured hindlimbs (7 days) showing PLOD2, MMP14, LOXL2. **i** Quantification of PLOD2^+^, MMP14^+^ and LOXL2^+^ cells in total cells; *n* = 3/group

Furthermore, micro-computed tomography (microCT) imaging was used to quantify bone formation in TSP1/2 DKO mice versus WT mice post-injury. Our B/T model predictably induces HO in four distinct anatomical regions: the calcaneus, tibia, distal residual Achilles tendon, and proximal residual tendon, nine weeks following injury.^8,9,23–32^ Compared to WT mice, TSP1/2 DKO mice exhibited a significant decrease in HO volume, with almost no HO formation observed in the distal and proximal residual tendons (Fig. 5a–d). No significant difference was observed between WT and DKO mice in tibial length or thickness in our model (Fig. S6A–D). Histological analysis of DKO mice revealed significant differences in tissue organization compared to control mice. In control mice, a distinct HO anlagen can be identified, characterized by PDGFRα^+^ cells surrounding the perimeter of the differentiating SOX9^+^ cells. In contrast, DKO mice did not exhibit this organized perimeter of PDGFRα^+^ cells, resulting in in a disrupted and disorganized distribution of dual positive PDGFRα^+^ SOX9^+^ cells at the HO anlagen (Fig. 5e–g). TSP1 and 2 signaling is critical for osteogenesis in our model and plays an essential role in HO formation in collagen-rich areas like tendon.

To investigate the molecular mechanisms underlying the disorganized ECM phenotype observed in TSP1/2 DKO mice, we first analyzed baseline transcriptional changes in the uninjured Achilles tendon. Bulk RNA-sequencing revealed downregulation of several matrisome-associated genes in DKO mice compared to WT, including *Thbs1, Thbs2*, (confirming knockdown) *Plod2, Spp1, and Alox12*, while expression of major fibrillar collagens remained unchanged (Fig. S6E). KEGG pathway enrichment of differentially expressed genes revealed suppression of ECM-receptor interactions and focal adhesions signaling pathways (Fig. S6F). *Spp1* (osteopontin) is a mineral-binding glycoprotein involved in early matrix remodeling and ossification.^41,42^ *Spp1* downregulation may reflect impaired osteogenic matrix priming and defective mineralization signaling. *Plod2* encodes lysyl hydroxylase 2, which catalyzes collagen crosslinking, and is essential for stabilizing newly deposited matrix during tissue repair and previously implicated in HO formation.^27^ *Alox12*, a lipoxygenase, modulates inflammation and redox signaling in the wound environment, and has been implicated in mesenchymal differentiation and tissue remodeling.^43,44^ together suggesting impairment in ECM stabilization and inflammatory remodeling pathways. To evaluate how this baseline deficit affects the injury response, we performed immunofluorescence staining for the collagen crosslinking enzymes LOXL2 and PLOD2, and the matrix-remodeling protease MMP14, at day 7 post-injury.^27,45–47^ All three markers were significantly reduced in DKO tissues compared to WT controls (Fig. 5h–j), indicating that TSP1 and TSP2 are required for proper upregulation of ECM remodeling enzymes during the early repair phase.

Together, these findings support a model in which thrombospondin signaling coordinates the structural organization of the early HO anlagen, enabling the proper orchestration of MPC differentiation and osteogenesis. In the absence of TSP1 and TSP2, ECM remodeling is impaired, resulting in a disorganized matrix that fails to support robust osteogenic differentiation, particularly along the Achilles tendon. Thus, *Thbs1* and *Thbs2* play essential roles in mature MPCs and the matrix environment required for ectopic bone formation following injury.

## Discussion

While a role for TSP1 and TSP2 in ECM structure and assembly has been demonstrated in uninjured models, this has not been explored in musculoskeletal injury models.^29,48,49^ Our study provides substantial evidence underscoring the critical role of TSPs in HO pathogenesis. By integrating single-cell RNA sequencing, spatial transcriptomics, and in vivo functional assays, we begin to elucidate the cellular mechanisms in which TSP1 and 2 participate in HO formation through early inflammatory mechanisms, ECM organization, and ultimately MPC osteochondral lineage fate following injury.

First, our results highlight the differential expression patterns of *Thbs1* and *Thbs2* in response to injury. *Thbs1* is upregulated in macrophages at the HO anlagen, particularly within M2-like macrophage populations is re-expressed in mesenchymal-progenitor cells (MPCs) at day 21, consistent with published reports that both thrombospondins are produced by osteolineage cells. This suggests a role for *Thbs1* in the inflammatory response and subsequent stages of tissue repair and endochondral ossification.^30,50,51^ Conversely, *Thbs2* expression is significantly elevated in MPCs around the HO anlagen, peaking one-week post-injury. This finding, validated by our TSP2eGFP reporter system, underscores the role of TSP2 in ECM remodeling and MPC differentiation. The presence of TSP2^+^ MPC cells with key chondrogenic and osteogenic markers such as SOX9 and OSX further supports its involvement in the early stages of osteogenesis within the HO microenvironment.^52,53^ Our spatial transcriptomic analyses provides a detailed map of TSP1 and 2 expressions in relation to tissue architecture and gene expression gradients. The distinct spatial expression patterns of *Thbs1* and *Thbs2* suggest specialized roles in different phases and regions of HO formation. The high expression of *Thbs1* at the HO site aligns with its role in facilitating macrophage-mediated HO formation.

In contrast, the peripheral expression pattern of *Thbs2* around the HO site and its association with collagen fibrillogenesis suggest a role in stabilizing and organizing the ECM to support bone formation.^48^ Furthermore, identifying FUBP1—a positive regulator of c-*Myc* that accelerates osteogenic gene expression—as a potential regulator of *Thbs2* underscores the significance of TSP2-mediated MPC osteogenic differentiation.^54–56^ While these findings across independent systems support a potential regulatory role for FUBP1 in controlling *Thbs2* expression, it is important to note that this relationship may be indirect or multifactorial. Given FUBP1s involvement in RNA processing, splicing, and transcriptional regulation, the observed reduction in *Thbs2* could reflect direct transcriptional control, altered splicing at the *Thbs2* locus, or secondary downstream effects.^40^ Additional mechanistic studies such as promoter assays or CHiP PCR are warranted to determine whether *Thbs2* is a direct transcriptional or post-transcriptional target of FUBP1. Further studies on FUBP1 regulation within injury models are necessary to clarify this potential relationship.

Previous studies demonstrated that TSP1 null mice exhibit altered responses to vascular injuries and impaired wound healing, indicating its role in tissue repair.^18,57,58^ TSP2 null mice have been shown to have impaired ECM organization and abnormal collagen fibrillogenesis highlighting TSP2’s crucial role in ECM integrity and repair mechanisms.^58–60^ Furthermore, TSP1/2 DKO mice exhibit significant skeletal abnormalities, including reduced bone formation, defects in bone remodeling, and compromised bone quality. These mice are known to have defects in collagen matrix maturation, overall underscoring the importance of TSP1 and TSP2 in maintaining bone homeostasis and proper ECM organization.^58,61–63^ The significant reduction in HO volume observed in TSP1/2 DKO mice further demonstrates the functional importance of TSP1 and TSP2 in ectopic bone formation. The absence of TSP1 and TSP2 disrupts ECM alignment and reduces osteogenesis, as evidenced by the decreased anisotropy in collagen fibrils and lower HO bone volume in DKO mice. These findings confirm the need for TSP1 and 2-mediated signaling to properly orchestrate cellular and molecular processes driving HO formation.

This study has several limitations. Primarily, our work focused on analyzing the spatial and temporal expression patterns of TSP1 and TSP2 in HO formation rather than conducting in-depth investigations into in vivo modifications or the detailed mechanisms of action for these proteins. While spatial transcriptomics and scRNAseq provided valuable insights into the localization and dynamic changes in TSP1 and TSP2 expression, understanding the mechanism of TSP1 and TSP2 influence on MPC cellular behavior in the context of HO is crucial for translating our findings into therapeutic strategies. While we used a double knockout model to investigate the impact of total TSP1 and TSP2 knockdown in our model, the utilization of lineage-specific knockouts will further tease out the importance of macrophage-derived TSP1 and MPC-derived TSP2 on ECM organization and osteogenesis. Future research will focus on elucidating the mechanism of TSP1 and 2 signaling in the context of HO. Investigating the interplay between TSPs and other key signaling molecules involved in ECM dynamics and osteogenesis will provide a more comprehensive understanding of HO pathogenesis. Additionally, exploring the role of TSPs in other models of HO and in human patients will be crucial for translating our findings into clinical applications.

Our study provides a foundation for identifying a novel TSP1 and 2 mechanism in ECM alignment and eventual HO formation following musculoskeletal injury and potential therapeutic interventions targeting TSP signaling pathways to prevent or mitigate HO. By defining the specific roles of TSP1 and TSP2 in key cell populations to HO formation, we can now explore targeted strategies to modulate their expression or function.

## Materials and methods

### Animal experiments

All mice studies were reviewed and approved by The University of Texas Southwestern Medical School Institutional Animal Care and Use Committee (Protocol 2023-103445) and the University of Michigan Institutional Animal Care and Use Committee (Protocol PRO00011260). Male and female mice between the ages of 8 and 12 weeks old underwent a 30% total body surface area back burn with concurrent Achilles transection as previously described.^8,9,24–27,30–32,64^

### Confocal microscopy and image analysis

Confocal microscopy images were obtained using a Leica Stellaris DMi8 microscope with an HC PL APO CS2 20x objective and a 3.15x digital zoom, achieving a 63x magnification for detailed analysis. Raw images for Hoechst and the antigen of interest were exported through Leica Application Suite software. A blinded, experienced operator analyzed the immunofluorescence images using CellProfiler software, applying consistent thresholding parameters across all experiments to quantify antigen expression and total cells.^65^ Mean and standard deviation were calculated and visualized using GraphPad Prism 8 (GSL Biotech LLC, Chicago, IL). For second harmonic generation (SHG) imaging, 20-µm tissue sections were imaged with a Zeiss LSM880 inverted confocal/multiphoton microscope, equipped with a forward SHG 400 to 480 nm M-2P filter and an 850-nm excitation laser with a transmitted light detector. Collagen fiber alignment was assessed using FibrilTool in ImageJ, which calculates anisotropy values based on eigenvectors of the Hessian matrix.^66^ Three to four regions of interest per image were selected to determine anisotropy values, providing insights into collagen fiber alignment.

### Histology and immunofluorescent staining

Hindlimb samples were harvested at 3 days, 1 week, 3 weeks, and 9 weeks following B/T injury. Legs were fixed in 4% paraformaldehyde (PFA) for 24 h at 4 °C, washed with 1 x PBS, and decalcified using 14% EDTA for 5-6 weeks until adequate decalcification was observed. Tissues were then embedded in optimal cutting temperature media to prepare longitudinal sections of frozen tissues. Sections were cut at 12 μm for immunofluorescence. For immunofluorescence staining, sections were thawed and then washed in 1 x tris-buffered saline with Tween 20. Sections were blocked with donkey serum blocking solution (1% bovine serum albumin, 2% donkey serum, 0.1% cold water fish skin gelatin, 0.05% Triton X-100, 0.05% Tween 20, 300 mmol/L glycine, and 1 x TBS) for 2 h at room temperature (RT). They were then incubated overnight with primary antibodies. Slides were washed in 1 x TBS-T three times and incubated with fluorescence-conjugated secondary antibodies. Then slides were stained for nuclei with Hoechst 33342 for 5 min, washed in 1 x TBS-T, and mounted with Prolong Glass Antifade Mountant and #1.5 Slip-Rite cover glass.

The following antibodies were used: anti-PDGFRa (1:100, goat monoclonal; R&D, AF1062), anti-SOX9 (1:100, rabbit monoclonal, Abcam, ab185230), and anti-OSX (1:100, rabbit monoclonal, Abcam, ab22552) and secondary antibodies conjugated with donkey anti-rabbit IgG H&L Alexa Flour 594 (1:1 000, Thermo Fischer Scientific, A-21207) and donkey anti-goat IgG H&L Alexa Flour 647 conjugated (1:1 000, Thermo Fisher Scientific, A-21447). Microscopic digital images of the immunofluorescence-stained sections were captured with 10x, 20x, and 40x objectives using upright fluorescent microscopy (Leica DM6, camera DFC3000G).

### Isolation of murine MPCs

Murine tissue-resident MPCs were isolated from tissue collected from the Achilles tendon injury site (soft tissue posterior to the tibia and in between the distal tendon enthesis to the calcaneus and proximal insertion to the gastrocnemius muscle) of mice as previously described.^8,23,25,29^ Briefly, harvested tissue were digested in digestion solution (3 mg/mL collagenase I, 2 mg/mL collagenase II, and 2 mg/mL dispase II), digested tissue was strained through a 40-μm cell strainer, and digestive enzymes were quenched in complete culture media [DMEM-10; Dulbecco’s modified Eagle’s medium (DMEM) supplemented with 10% FBS (Gibco)] and 1x antibiotics [penicillin (100 U/mL) and streptomycin (100 g/mL)]. Cells were spun down at 300 *g* for 5 min, and the supernatant was discarded. The cell pellet was subsequently explained in complete media and passages with split 1:3 ration. All cells were used within passages 1 to 4.

### MicroCT imaging and analysis

Whole hindlimb samples from subject mice were harvested at 9 weeks following B/T injury. Legs were fixed in 4% paraformaldehyde (PFA, in PBS) for 18 h at 4 °C, and washed with 1 x PBS. MicroCT images were taken at the University of Texas Southwestern Department of Radiology using a nanoScan PET/CT system (Mediso USA). Images were quantified using Dragonfly (Object Research Systems) at 800 Hounsfield units (HU). Reconstructions of mouse hindlimbs were created on representative means at 800 HU. Quantifications at 800 HU of ectopic bone formation were performed on four anatomical regions: calcaneus, tibia, distal residual Achilles tendon, and proximal residual Achilles tendon. A blind, skilled operator quantified the area of ectopic bone. Quantifications of total ectopic bone formation included all four of these regions. Tendon HO is defined by ectopic bone in the distal and proximal residual Achilles tendon. Average cortical thickness of the tibia and tibial length were also quantified in these 3D reconstructions. Tibial length was defined as the length between the middle of the proximal head to the middle of the distal end of the tibia.

### Single-cell RNA sequencing

ScRNAseq for days 0, 3, 7, and 21 days after B/T injury was previously published (GSE12606).^31^ Briefly, approximately 500 million reads were generated per replicate using 10× Genomics sequencing. The data was pre-processed with Cell Ranger (10× Genomics, Pleasanton, CA, USA) to align reads against the mm10 genome, achieving 94% alignment and a median of 2 600 genes per cell. Downstream analysis was performed using the Seurat R package. Cells with fewer than 500 genes or more than 25% mitochondrial read content were filtered out. Subsequent analysis included normalization, identification of highly variable genes, scaling based on UMI counts and batch effects, dimensionality reduction (PCA, t-SNE), unsupervised clustering, and differential expression analysis. Canonical correlation analysis (CCA) in Seurat was used to compare datasets, aligning and clustering cells from different sample types. Differentially expressed genes between aligned clusters were identified using a negative binomial test. Pseudotime analysis of MPCs was performed using Monocle3 to identify the differentiation trajectory, with automated root cell identification.^67^ Transcription factor (TF) binding motif enrichment analysis was conducted using the RcisTarget package (v1.8.0) to identify TFs potentially regulating *Thbs2* within MPCs. The *Thbs2* covariant gene set, derived from Monocle3 (v1.0.0) trajectory analysis, was used to assess ECM-modulating cells. Motif rankings were imported from the mm10_10kbp_up_10kbp_down_full_tx_v10_clust.genes_vs_motifs.rankings.feather database provided by RcisTarget, containing motif data centered around transcription start sites (TSS). Significant motifs linked to *Thbs2* module genes were identified based on area under the curve (AUC) scores and enrichment thresholds. The motifAnnotations_mgi dataset was applied for mouse gene symbol annotation, and FUBP1 was identified as a highly enriched TF in the gene set. The FUBP1 gene regulatory network was generated using the STRING database in Cytoscape (v3.10.3) via stringApp (v2.1.1).

ScRNAseq data for Blood, Bone Marrow and Injury Site 3- and 7-days post HO, was obtained from GEO (GSE221134 and GSE12606 respectively).^26^ All computational analysis was performed using the Seurat R package (v5).^68^ Genes expressed in less than three cells and cells expressing fewer than 200 genes were removed by primary filtering. After quality control, cells expressing more than 3% mitochondrial reads in all genes were excluded. The read counts per cell were normalized and scaled. All these datasets were merged, and SC Transformation was performed. Then we did principal component analysis and retained the first 50 principal components. It was followed by Harmony Integration and clustering the cells with a resolution of 0.2. The FeaturePlot function was used to visualize single cells on a UMAP plot according to gene expression. The marker genes for each cluster were identified by a Wilcoxon rank-sum test, with an adjusted *P* value < 0.05 by Bonferroni correction.

### siRNA knockdown of *Fubp1*

Tendon MPCs were seeded into 6-well plates at a density of 500 000 cell/well in complete growth medium and cultured to approximately 70%-80% confluence. For siRNA transfection via electroporation, cells were harvested by trypsinization, counted, and resuspended in P3 Primary Cell Nucleofector Solution. Each reaction contrained 100 nmol/L siRNA. The following siRNAs were all purchased from Invitrogen: Silencer^TM^ Selected Negative Control siRNA and Silencer^TM^ Select *Fubp1* siRNA s78693. The cell/siRNA mixture was transferred to a certified electroporation cuvette and electroporated using Lonza 4D Nucleofector. Immediately after electroporation, the cells were gently transferred into pre-warmed complete growth medium in 6-well plates. Cells were incubated at 37 °C in a humidified incubator with 5% CO₂. Culture medium was replaced 8 h later to remove any residual transfection reagents. Knockdown efficiency was assessed 48–72 h post-transfection by quantitative PCR.

### Spatial transcriptomics

Previously deposited spatial transcriptomics data was reanalyzed for this study (GSE255942) (PMID: 38472175). Downstream data analysis and visualizations were performed using Seurat 4 (PMID: 37231261). Using the integrated scRNAseq data as reference, prediction values were calculated for MPC and myeloid cell populations at each spatial spot. Spots were identified as cell-type positive or negative using a cutoff of 0.6, before gene expression analysis was performed. Distance scoring (Spatial Time) was performed as done previously (PMID: 37926705). Briefly, the HO anlagen was manually marked. Distance was calculated for each spatial transcriptomics spot with respect to each pixel of the marked HO anlagen. Distances were scaled to distances between 0, referring to close to the injury site, to 1, referring to farther position from the anlagen site. Gene expression plots were generated by binning distance scores at 0.01 intervals, and calculating the average expression and standard error for each bin. A smoothed line of binned averages was visualized in black, average expression per bin was visualized in a blue solid line, and standard error was visualized in a blue dotted line (Prism GraphPad).

### Statistical analyses

Statistics were performed using GraphPad Prism 9 (San Diego, CA). All comparisons were performed at α = 0.05. Tests for normality and heterogeneity of variance were performed to ensure appropriate statistical tests were performed. When necessary, Welch’s correction was performed on heteroscedastic data and Mann-Whitney *U*-Tests were performed for non-parametric data. Statistical significance is displayed on each graph as an asterisk (*) for *t*-test and a pound (#) for Mann-Whitney *U*-Test. In experiments with multiple groups or treatments, statistical significance is displayed on each graph as an asterisk (*) for ANOVA test.

## Supplementary information

Supplementary Material

## Data Availability

All data needed to evaluate the conclusions in the paper are present in the paper and/or the Supplementary Materials. Data from the Day 7 blood and bone marrow sequencing experiment are available at NCBI GEO, and additional bioinformatics analyses were performed on previously published data.
