# Supplementary material for: Thrombospondin 1 and 2 regulate mesenchymal progenitor cell fate and matrix organization
Source: Bone Res. 2026 Jan 19;14:10. doi: 10.1038/s41413-025-00493-2 (PMC12816047; doi:10.1038/s41413-025-00493-2)
Supplement: Supplementary file 1 — Supplementary Material [file 41413_2025_493_MOESM1_ESM.docx]

**SUPPLEMENTARY MATERIAL**

**
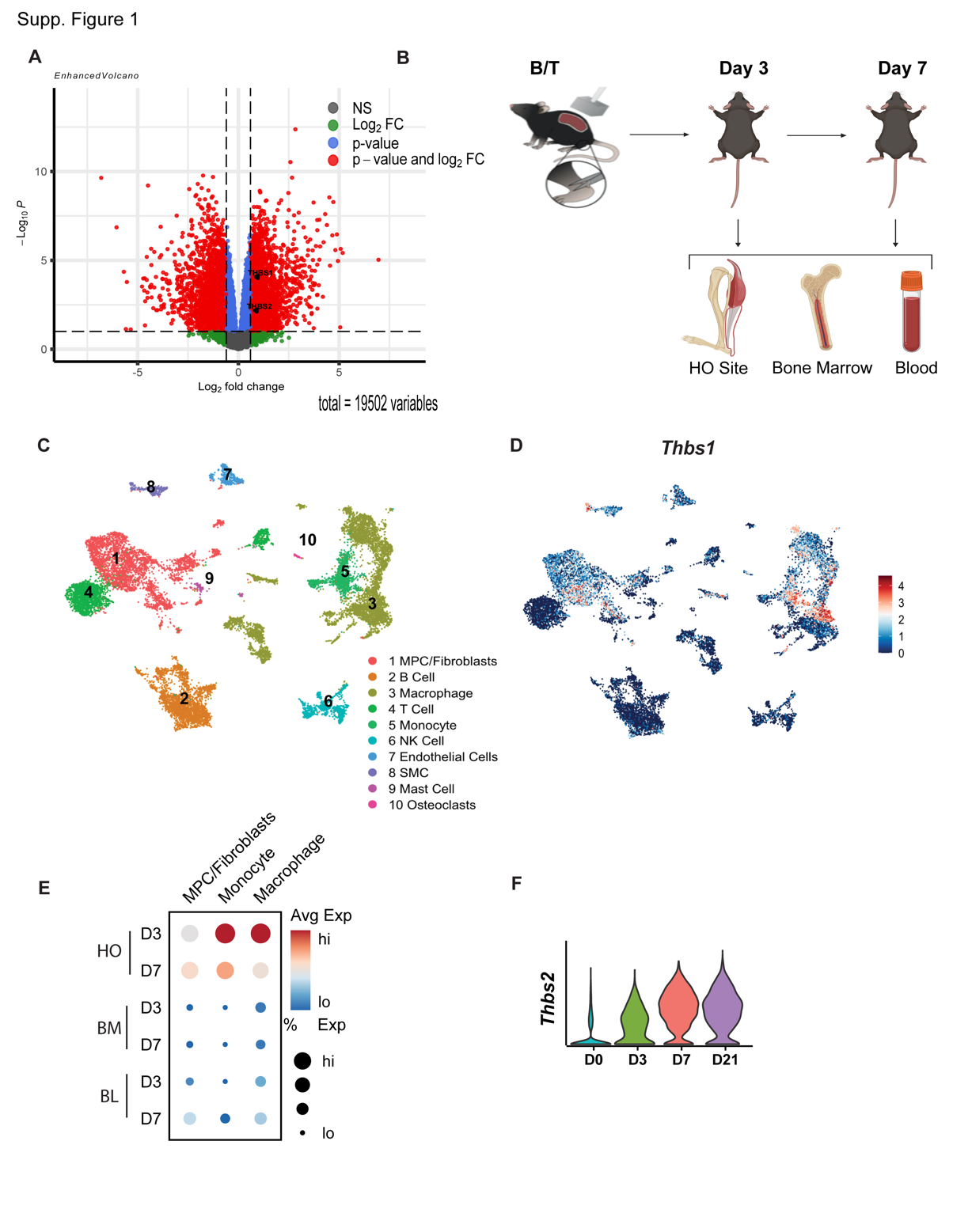
**

**Fig. S1.** ***Thbs1* & *Thbs2* expression in other cell types.** **(A)** Volcano plot of differential gene expression between bone marrow MPCs from patients with neurogenic HO and healthy donors created using ‘limma’ and the R package ‘EnhancedVolcano.’ *THBS1* logFC=0.892521, p=0.0001013622; *THBS2* logFC=0.844618, p=0.0077418202 **(B)** Schematic illustrating scRNA seq collection of HO site, bone marrow (BM) and blood (BL). **(C)** UMAP for cells harvested from BM, BL, and HO injury site 3 and 7 days after B/T injury. **(D)** Feature plot of *Thbs1*. **(E)** Dot plot of *Thbs1* across day 3 and day 7 at the HO site, BM, and BL in MPC/Fibroblasts, Monocytes, and Macrophage clusters. **(F)** Violin plot of *Thbs2* expression in cells from injured mice at days 0, 3, 7, and 21**.**


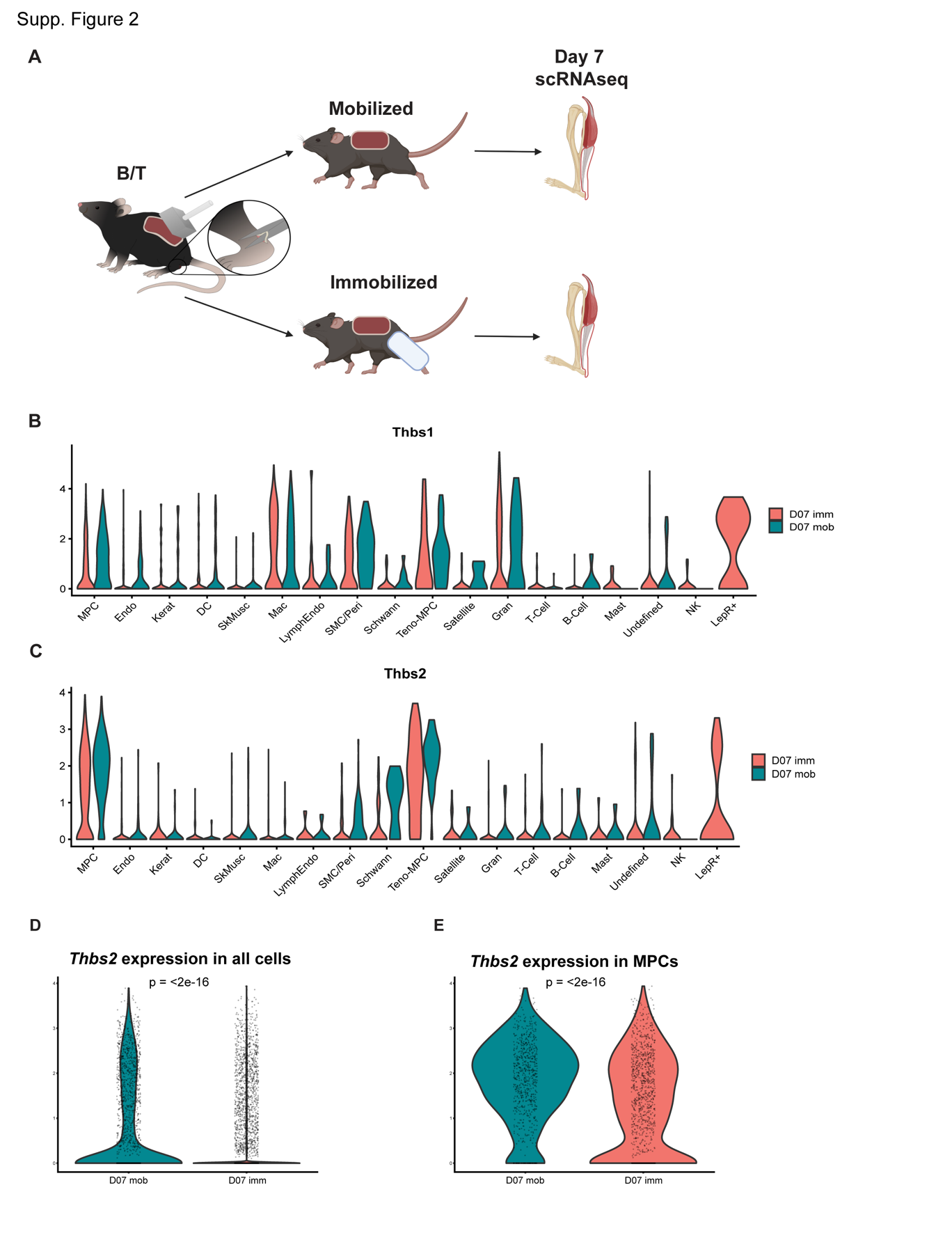


**Fig. S2.** ***Thbs1* and *Thbs2* expression in immobilized mice. (A)** Schematic of mobilized versus immobilized tissue collected for scRNA seq**. (B)** Violin plots of *Thbs1* expression in cells collected from the injury site 7 days after B/T in mobilized vs immobilized mice across all clusters. **(C)** Violin plots of *Thbs2* expression in cells collected from the injury site 7 days after B/T in mobilized vs immobilized mice across all clusters. **(D)** *Thbs2* expression in all cells in day 7 mobilized mice vs day 7 mobilized mice. **(E)** *Thbs2* expression in MPC cluster in day 7 mobilized vs day 7 immobilized mice.


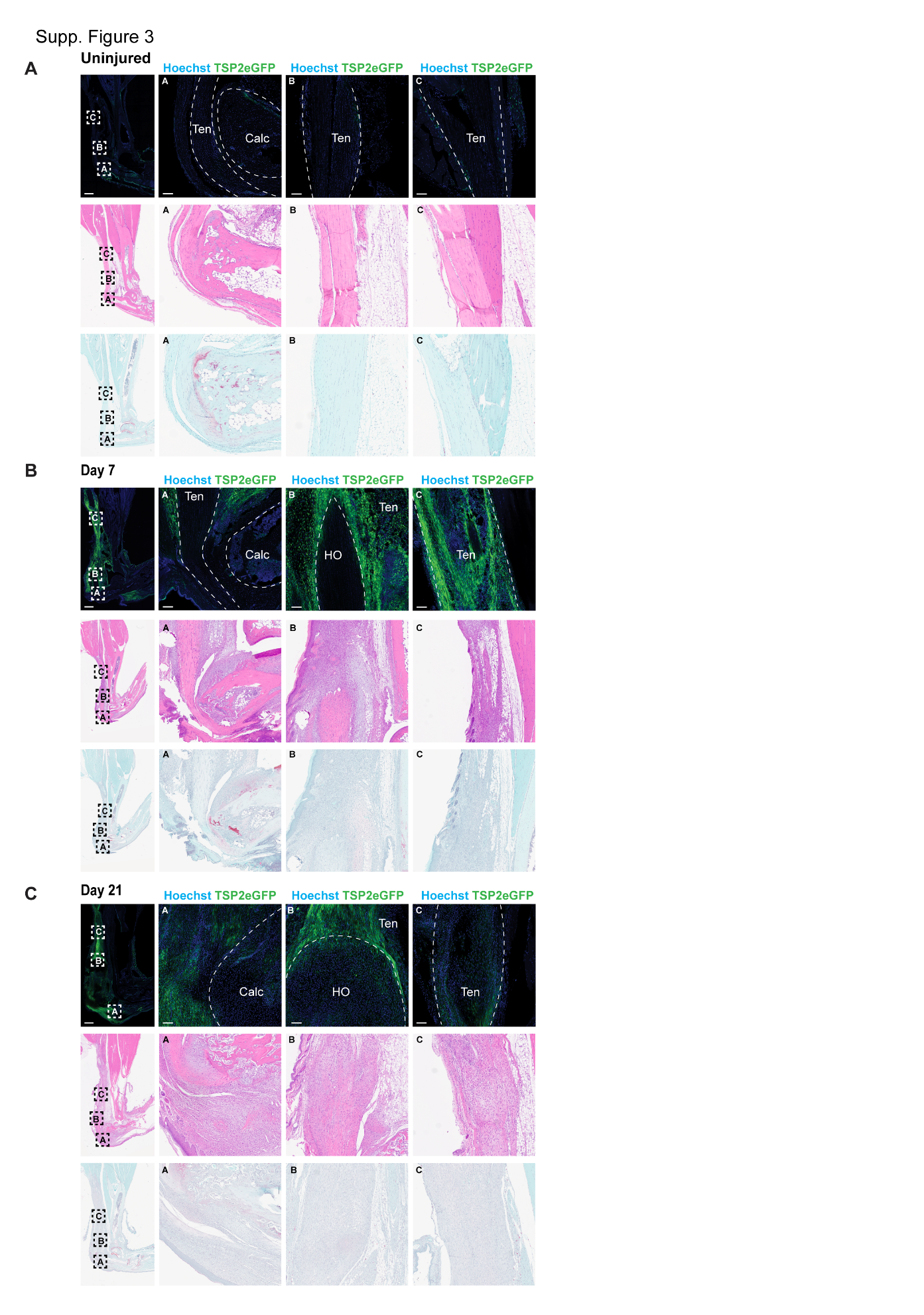
**Fig. S3** **Multimodal histological orientation of the injury site using TSP2-eGFP immunofluorescence, hematoxylin and eosin (H&E), and Safranin O staining across key time points post-injury. (A)** Uninjured (Day 0) hindlimb tissue showing baseline anatomy and ECM architecture in the Achilles tendon region, including TSP2eGFP signal (green), H&E staining for general morphology, and Safranin O to evaluate proteoglycan distribution. **(B)** Day 7 post-injury shows increased TSP2eGFP+ cells, matrix disorganization, and early proteoglycan deposition, consistent with early repair. **(C)** Day 21 reveals persistent TSP2-eGFP expression, disrupted tendon architecture, and enhanced Safranin O staining within the forming HO lesion. Magnified panels correspond to boxed regions from full tile scans


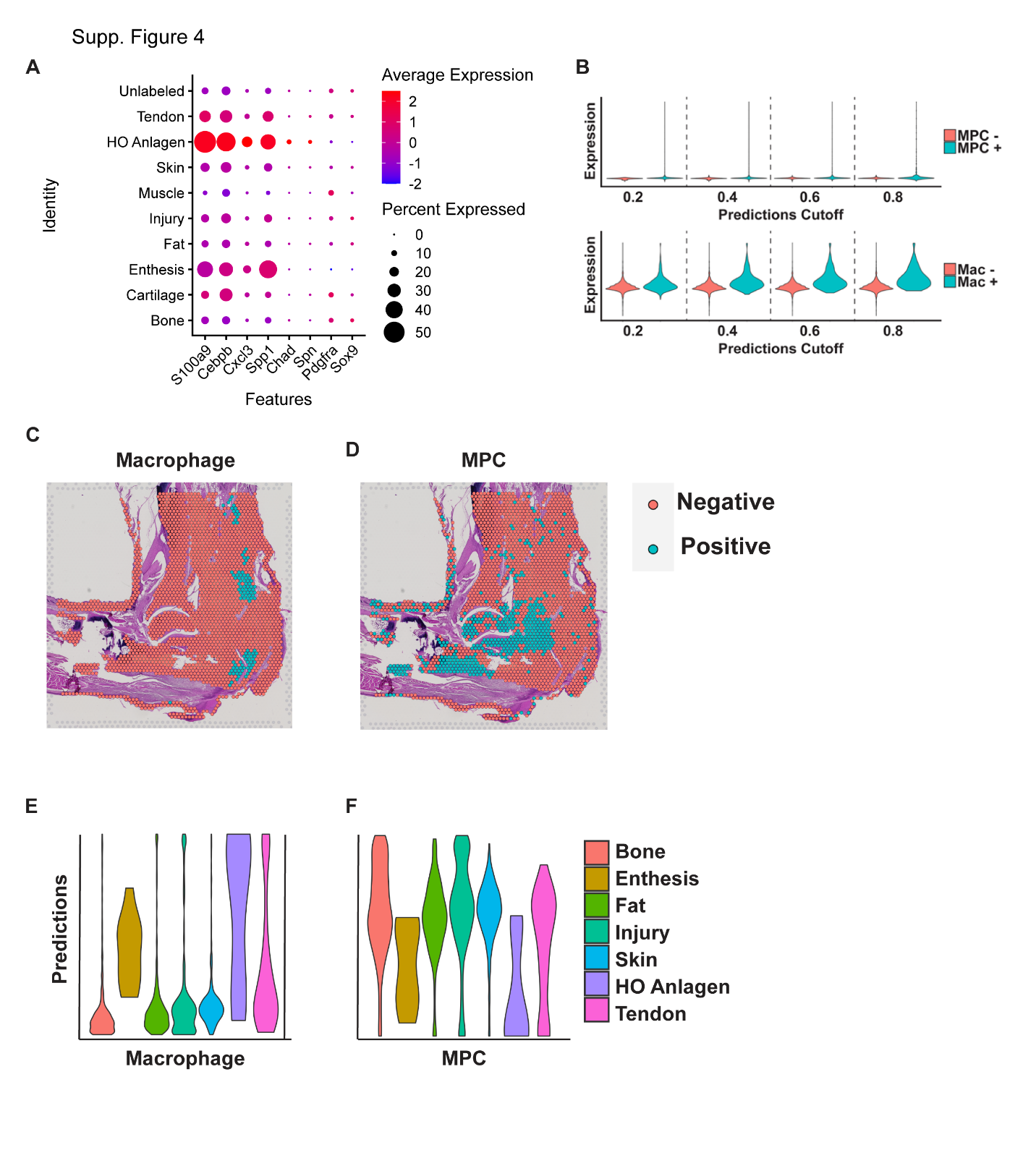


**Fig. S4.** **Spatial Transcriptomics of cell types within and surrounding the HO anlagen. (A)** Dot plot of key DEGs in the HO anlagen across all segmentation clusters. **(B)** Violin plots showing expression of the top 75 DEGs for mesenchymal progenitor cells (MPCs, top) and macrophages (Macs, bottom) across varying prediction score thresholds (0.2–0.8) in the spatial transcriptomic dataset **(C)** Spatial spots determined as negative or positive for macrophage cell types. **(D)** Spatial spots determined as negative or positive of MPCs. **(E)** Violin plot of macrophage prediction scores across anatomical sites. **(F)** Violin plot of MPC prediction scores across anatomical sites.


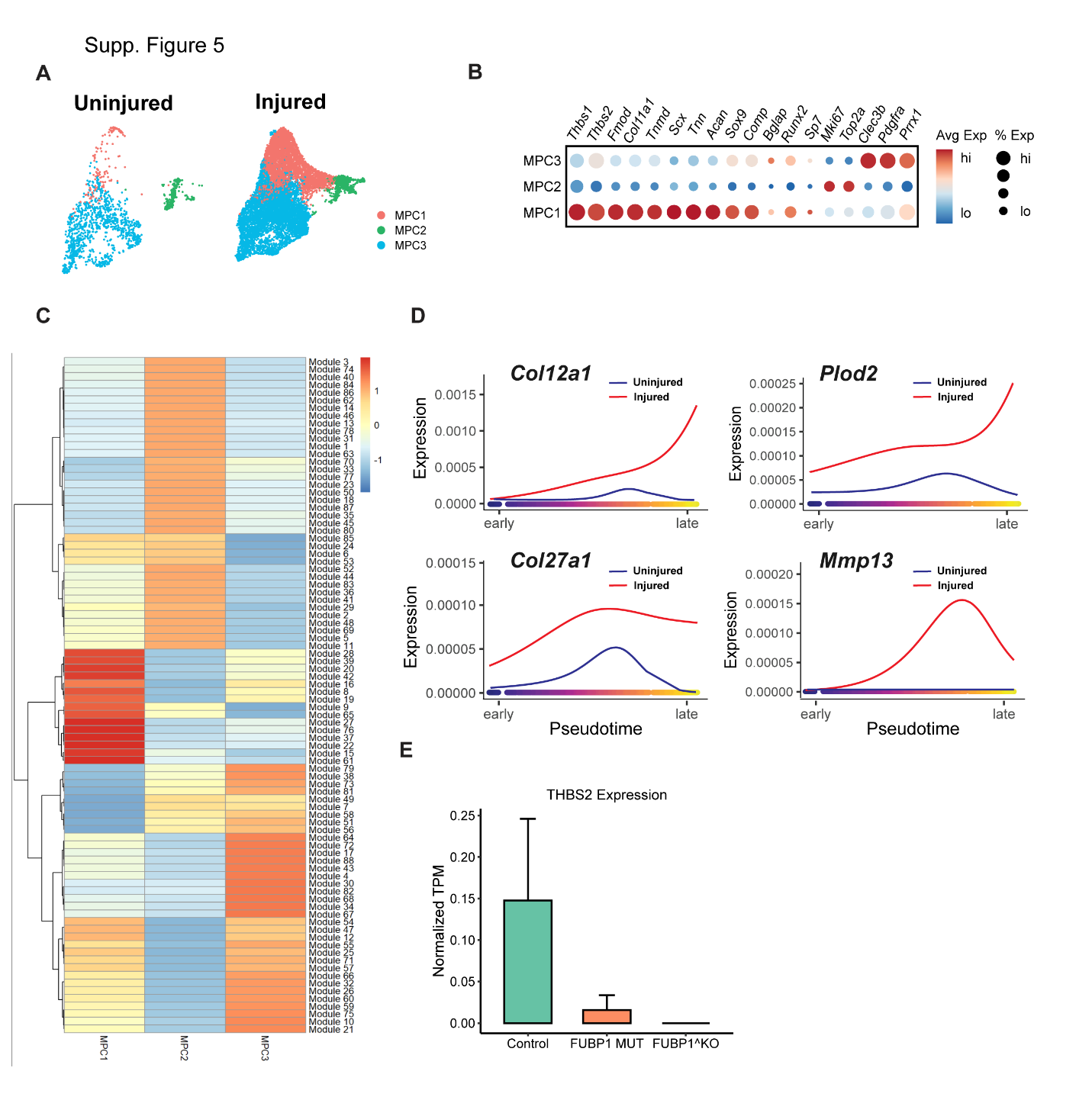


**Fig. S5.** **MPC trajectory analysis and covariant gene regulatory network. (A)** Umap of Pseudotime analysis clustering in MPCs collected from uninjured and injured mice at the injury site. MPC1 is defined as ECM remodeling, MPC2 proliferative, and MPC3 general. **(B)** Dot plot of genes used for identifying cell clusters in A. **(C)** Heatmap of co-regulated gene modules during MPC differentiation**. (D)** Pseudotime trajectory regression in uninjured (blue) and injured (red) MPCs for covariant genes. **(E)** Bar graph of Thbs2 expression in control, FUBP1 knockout, and FUBP1 mutant cells.


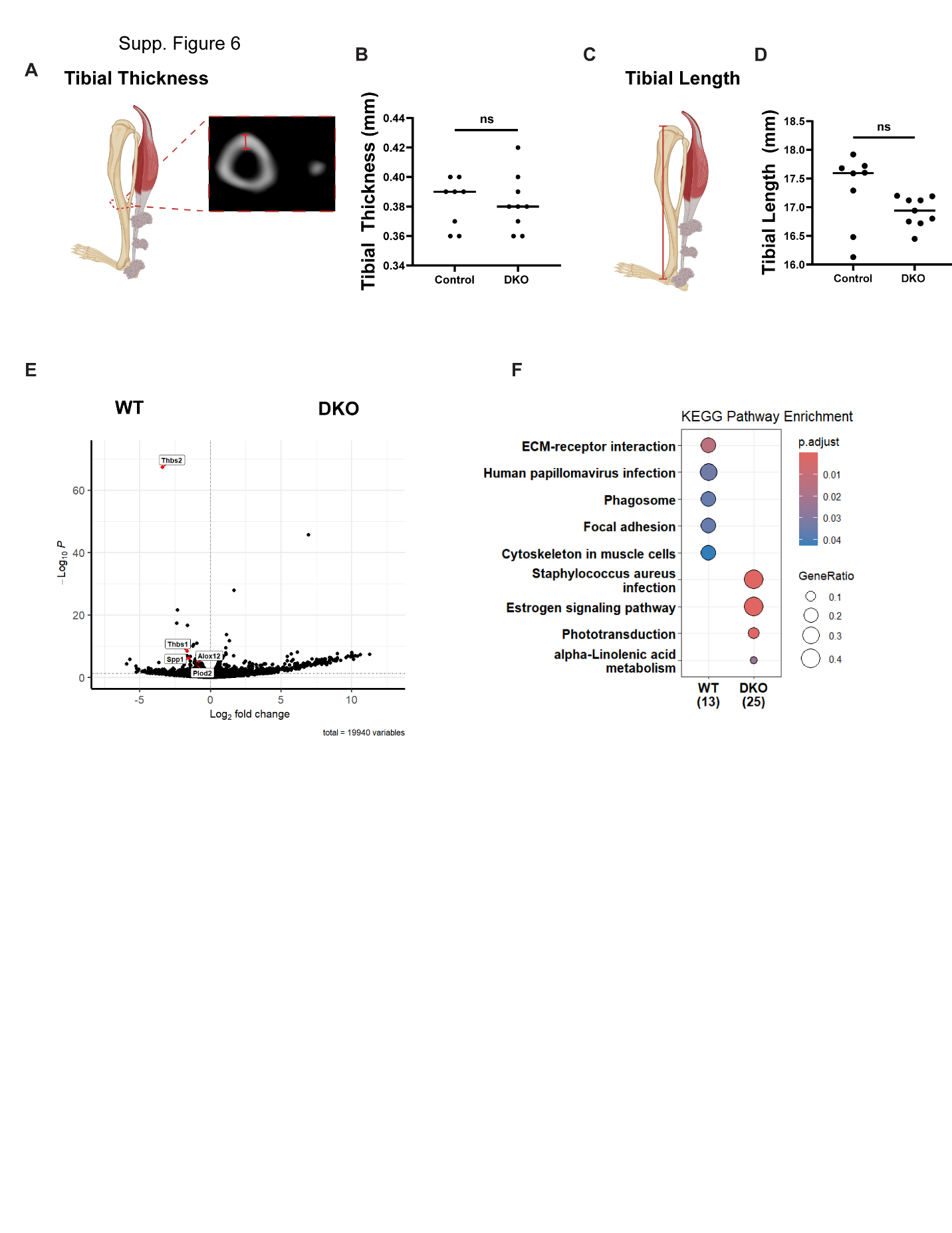


**Fig. S6.** **TSP1/2 DKO mice compared to WT at baseline (A)** Schematic of tibial thickness calculation. Solid red line indicates thickness. **(B)** Quantification of tibial thickness between control and DKO mice. **(C)** Schematic of tibial length calculation. Solid red line indicates length measurement. **(D)** Quantification of tibial length between control and DKO mice. **(E)** Volcano plot showing differentially expressed genes between WT and DKO tendons. Genes labeled include Thbs1, Thbs2, Plod2, Spp1, and Alox12, which are significantly downregulated in DKO tissue. The x-axis represents log₂ fold change; the y-axis shows the –log₁₀(p-value). **(F)** KEGG pathway enrichment analysis of significantly upregulated genes in WT and DKO groups. Each dot represents a pathway, with size indicating gene ratio and color corresponding to adjusted p-values.
